# Complete mitochondrial genome of an oleaginous microalga *Vischeria punctata* (Eustigmatophyceae: Chlorobotryaceae) and phylogenetic analysis

**DOI:** 10.1080/23802359.2023.2301027

**Published:** 2024-01-18

**Authors:** Zhouwei Luo, Zihao Wang, Yanhang Tang, Yuexin Sun, Yu Jiang, Wenjie Yang, Ge Chen, Luodong Huang

**Affiliations:** College of Life Science and Technology, Guangxi University, Nanning, China

**Keywords:** *Vischeria punctata*, oleaginous microalga, mitochondrial genome, Eustigmatophyceae, phylogenetic analysis

## Abstract

*Vischeria punctata*, as first described by Vischer in 1945, is a member of the family Chlorobotryaceae, within the order Eustigmatales. This species is recognized for its potential as a source of biofuels and other high-value products. In the present investigation, the whole genome of *V. punctata* was sequenced utilizing the Illumina HiSeq 4000 platform, enabling the assembly and annotation of its complete mitochondrial genome. The resulting circular genome spans 41,528 base pairs (bp) with a guanine-cytosine (GC) content of 27.3%. This genome encompasses 36 protein-coding genes, alongside 28 transfer RNA (tRNA), and three ribosomal RNA (rRNA) genes. The evolutionary trajectory of *V. punctata* was further explored by constructing a phylogenetic tree derived from the mitochondrial 33 gene dataset of 16 Ochrophyta species. Comparative analysis reveals that *V. punctata* bears closer ties to *Vischeria* sp. CAUP Q202 than to *Vischeria stellata* strain SAG 33.83, suggesting shared evolutionary pathways and phenotypic traits. This investigation constitutes the inaugural study into the mitochondrial evolution and phylogenetic patterning of the mitogenome in *V. punctata*. The outcomes from this research bolster our understanding of the genetic diversity and evolutionary processes within the class Eustigmatophyceae. In particular, the mitochondrial genome of *V. punctata* serves as a valuable resource in elucidating these aspects.

## Introduction

Biodiesel presents a compelling frontier in the sphere of renewable energy resources, with algae emerging as a crucial contributor to bioenergy production. One lineage within the Ochrophyta, the Eustigmatophyceae, consists of single-celled coccoid microalgae (Eliáš et al. 2017). Given their high lipid content in dry weight and rapid growth rate, species within the Eustigmatophyceae class have garnered attention as viable candidates for industrial biofuel microalgae (Jagadevan et al. 2018). While a substantial volume of research has centered on the *Nannochloropsis* and *Microchloropsis* genera from the Monodopsidaceae family – known for their robust lipid accumulation capabilities (exceeding 50% of dry weight) – less emphasis has been placed on exploring other Eustigmatophyceae species (Yang et al. 2021).

*Vischeria punctata* Vischer 1945, an oleaginous microalga from the Chlorobotryaceae family, is previously identified by Hibberd (1981) as belonging to Eustigmataceae family, and is another lineage within the Eustigmatophyceae class that has recently attracted substantial scientific interest (Barcytė et al. 2022). Notably, *Vischeria* sp. CAUP H4302, initially named as *Eustigmatos* cf. *polyphem*, exhibits remarkable lipid and biomass accumulation capabilities, achieving high biomass concentrations of 4.72 g/L and lipid contents of 71.45% of dry weight (Wang et al. 2018). Similarly, lipid contents of *Vischeria stellata* strain SAG 33.83 range between 55.9% and 66.79% of dry weight, with neutral lipids constituting 88–93% of this fraction, thereby rendering it a promising resource for biodiesel production (Gao et al. 2016; Wang et al. 2018).

Additionally, *Vischeria* sp. WL1, a novel strain of Eustigmatophyceae isolated from a large biological soil crust in north-western China, is characterized by high levels of C16:1 fatty acid and EPA (She et al. 2022). Furthermore, Eustigmatophyceae alga IPPAS H-242 was identified as a strain of *Vischeria punctata* based on 18S rRNA gene and ITS1-5.8S-ITS2 sequences (Sinetova et al. 2021). This strain exhibits an accumulation of eicosapentaenoic acid (EPA) under saline stress conditions, suggesting that *V. punctata* might be a valuable source of EPA. The hyper-accumulation of lipid by *Vischeria* signifies its potential for biodiesel and bioactive fatty acid production.

While numerous investigations have explored and optimized the cultivation conditions for *Vischeria* to yield enhanced biomass and lipid output, genomic studies are rather scant. The genomes of 12 strains across six species of *Nannochloropsis* and *Microchloropsis* have been sequenced, albeit few assemblies have attained significant contig continuity and completeness (Yang et al. 2021). A recently published study conducted the sequencing and assembly of two high-quality genomes of *Vischeria* sp. H4302 and *V. stellata* strain SAG 33.83, unveiling the potential molecular mechanisms underpinning the enhanced lipid and fatty acid accumulation ability, as well as high biomass production performance of *Vischeria* (Gao et al. 2023).

Unlike the complex and challenging assembly process of nuclear genomes, mitochondrial and chloroplast genomes have simpler structures, which can provide valuable phylogenetic information about the algae organism (Ševčíková et al. 2016; Huang et al. 2019). In this study, the mitochondrial genome of *V. punctata* was successfully sequenced and assembled. Following the annotation of the mitochondrial genome and the construction of a corresponding circular map, a phylogenetic analysis involving 16 Ochrophyta species was conducted to explore the evolutionary relationships of *V. punctata*. These findings pave the way for further molecular biology studies on this microalga, offering insights into evolutionary relationships within the Eustigmatophyceae class.

## Materials and methods

### Algae materials

**Figure 1.**
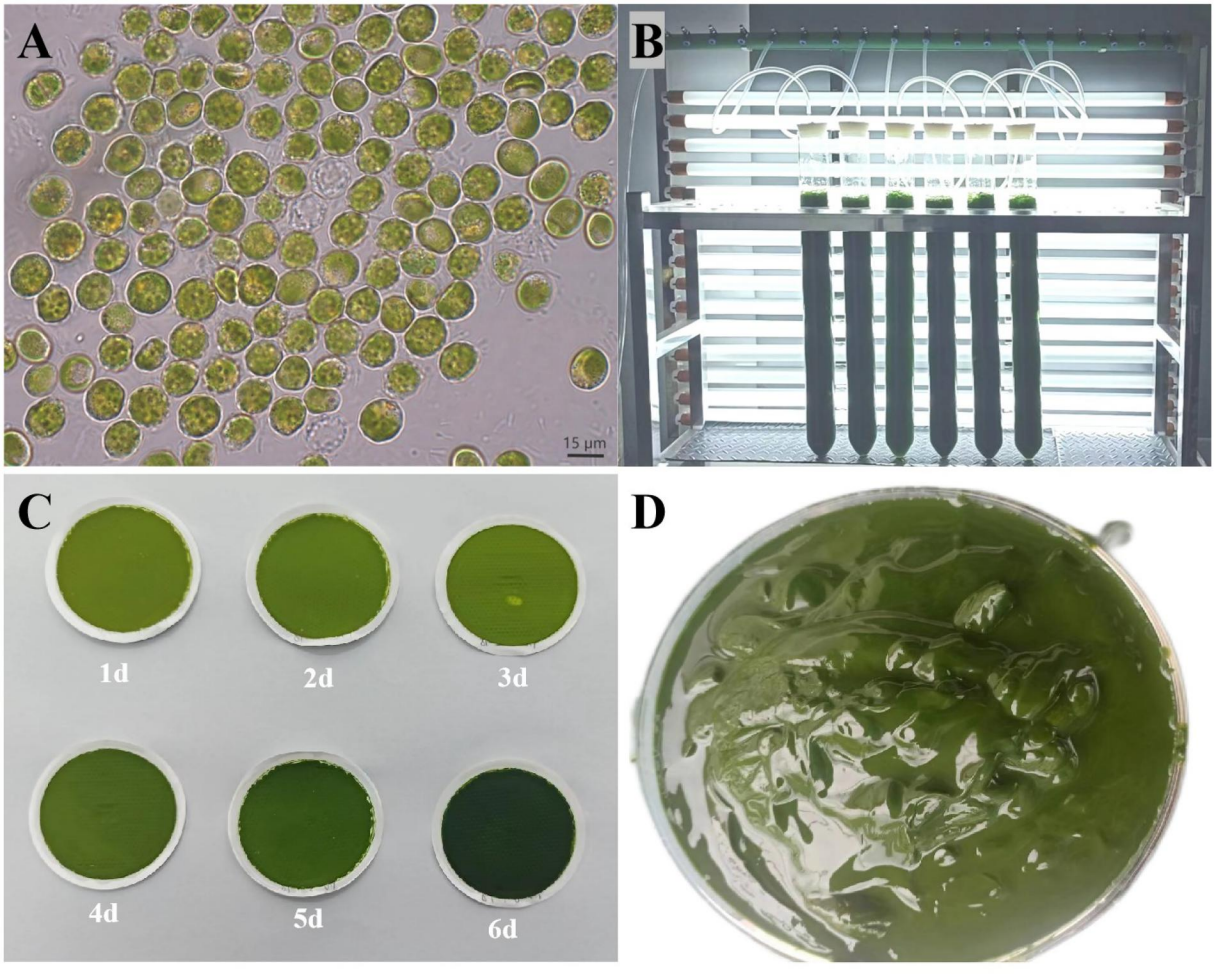
Algal characteristics of *V. punctata*. Panel A presents the morphological observations of cells under optical microscopy. The cells appear globular or angular, some with small humps, single parietal, much-lobed chloroplast and a single protruded polyhedral pyrenoid. Mature vegetative cells often reveal a cytoplasmic lipidic reddish globule. Panel B portrays *V. punctata* cultivated in bubble column glass photobioreactors. Panel C illustrates the color change of cells over time, from yellow-green on day one to dark green on day six. Panel D demonstrates *V. punctata* cells harvested on day six, which were used for DNA extraction in this study. These images were captured by Luodong Huang on 12 September 2020, at the Biological Resources and Environmental Microbiology Laboratory of the College of Life Science and Technology, Guangxi University.

The strain of microalga *Vischeria punctata* (UTEX 153) employed in this study was originally isolated from Basel, Switzerland (46.6631°N, 10.2413°W) and procured from the American Type Culture Collection (ATCC). The voucher specimen of *V. punctata* cells was cultivated in BG-11 medium (Figure 1(A)) and is deposited in the Herbarium of the College of Life Science and Technology, Guangxi University, Nanning, Guangxi, China (Dr. Luodong Huang, ynhuangld@gxu.edu.com), under voucher number GXU-A18.

### Microalgae cultivation, morphological observation, and DNA extraction

*V. punctata* was grown in a bubble column glass photobioreactor (Ø6 × 60 cm) containing 800 mL of mBG-11 medium (Figure 1(B)) and was exposed to continuous illumination of 200 μmol m^−2^ s^−1^ provided by fluorescent light for a period of six days (exponential growth phase). The cultivation temperature was maintained at 24 °C. Morphological examination was conducted using light microscopy based on the method delineated by Gao et al. (2016). Daily observation of the cells under a light microscope was carried out. Harvesting of the algal culture cells was performed by centrifugation at 3000 rpm for 5 min (Figure 1(D)), followed by storage in a freezer at −80 °C. Genomic DNA from *V. punctata* was isolated using the MiniBEST Plant Genomic DNA Extraction Kit (TaKaRa, Beijing, China) according to the manufacturer’s instructions. The DNA quality and concentration were determined using agarose gel electrophoresis and a NanoDrop One UV–Vis spectrophotometer (Thermo Fisher Scientific, Waltham, MA).

### Genome sequencing, assembly, and annotation of mitochondrial genome

The extracted genomic DNA was stored at −80 °C in Guangxi University, and the purified DNA was employed to construct a short genomic library. A 350 base pairs (bp) library was built for preparing a sequencing library with a TruSeq Nano DNA LT Sample Preparation Kit (Illumina, San Diego, CA), and paired-end sequencing was performed on an Illumina HiSeq 4000 platform provided by BGI Biotechnology Co., Ltd. (Shenzhen, China). The assembly and annotation of the mitochondrial genome were performed following the methodology of Abuduaini et al. (2021). The raw reads were pruned using Fastp, and the clean reads were utilized for the assembly of the mitochondrial genome using SPAdes 3.9.0 (Bankevich et al. 2012) with default settings. Following assembly, one contig of the mitochondrial DNA was visualized using Bandage software (Wick et al. 2015) and confirmed by aligning the sequences to the mitogenome of *V. stellata* strain SAG 33.83 (MH981596) (Huang et al. 2019). Subsequently, the annotation of the mitochondrial genome was executed using online tools, including MITOS (Bernt et al. 2013), MITOFY (Alverson et al. 2010), GeSeq (Tillich et al. 2017), tRNAscan-SE (Chan and Lowe 2019), RNAmmer v1.2 (Lagesen, et al. 2007), and UGENE ORFs finder (Okonechnikov et al. 2012). The annotation was then manually corrected using Apollo software (Dunn et al. 2019) and UGENE toolkit (Okonechnikov et al. 2012). The complete mitogenome of *V. punctata* was submitted to NCBI (GenBank accession no. MZ517137). The circular genome images and cis-splicing gene images, were generated using ogview (http://www.1kmpg.cn/ogview).

### Phylogenetic analysis

Fifteen mitochondrial genomes from Ochrophyta species were selected for phylogenetic analysis, with *Synura synuroidea* (AF222718) and *Ochromonas danica* (AF287134) designated as the outgroup. The common genes of mitochondrial genomes were extracted, concatenated, and aligned using PhyloSuite (Zhang et al. 2020), HomBlocks (Bi et al. 2018), and MAFFT (Katoh et al. 2002). The ModelFinder module of IQ-TREE (Nguyen et al. 2015) was adopted to get the optimum evolution model of mitochondrial genomes data rapidly. The optimum nucleotide substitution model was shown as GTR + F + I + G4 according to BIC standard. Ultimately, the phylogenetic tree was constructed based on the maximum-likelihood (ML) method using IQ-TREE (Nguyen et al. 2015), assisted by GTR + F + I + G4 models, with 1000 bootstrap replicates.

## Results and discussion

The microalga *Vischeria punctata* demonstrated distinctive traits characteristic of the *Vischeria* genus, such as an almost elliptical appearance, the presence of reddish globules within the cell, and a small proportion displaying humps on the cell wall (Figure 1(A)). The cell color predominantly began as yellow-green, and as the culture progressed, gradually transformed to green, dark green, and finally, an intense dark green (Figure 1(C)). On day six, which belongs to the exponential growth phase, algal cells were gathered through centrifugation for the purpose of water extraction (Figure 1(D)).

**Figure 2.**
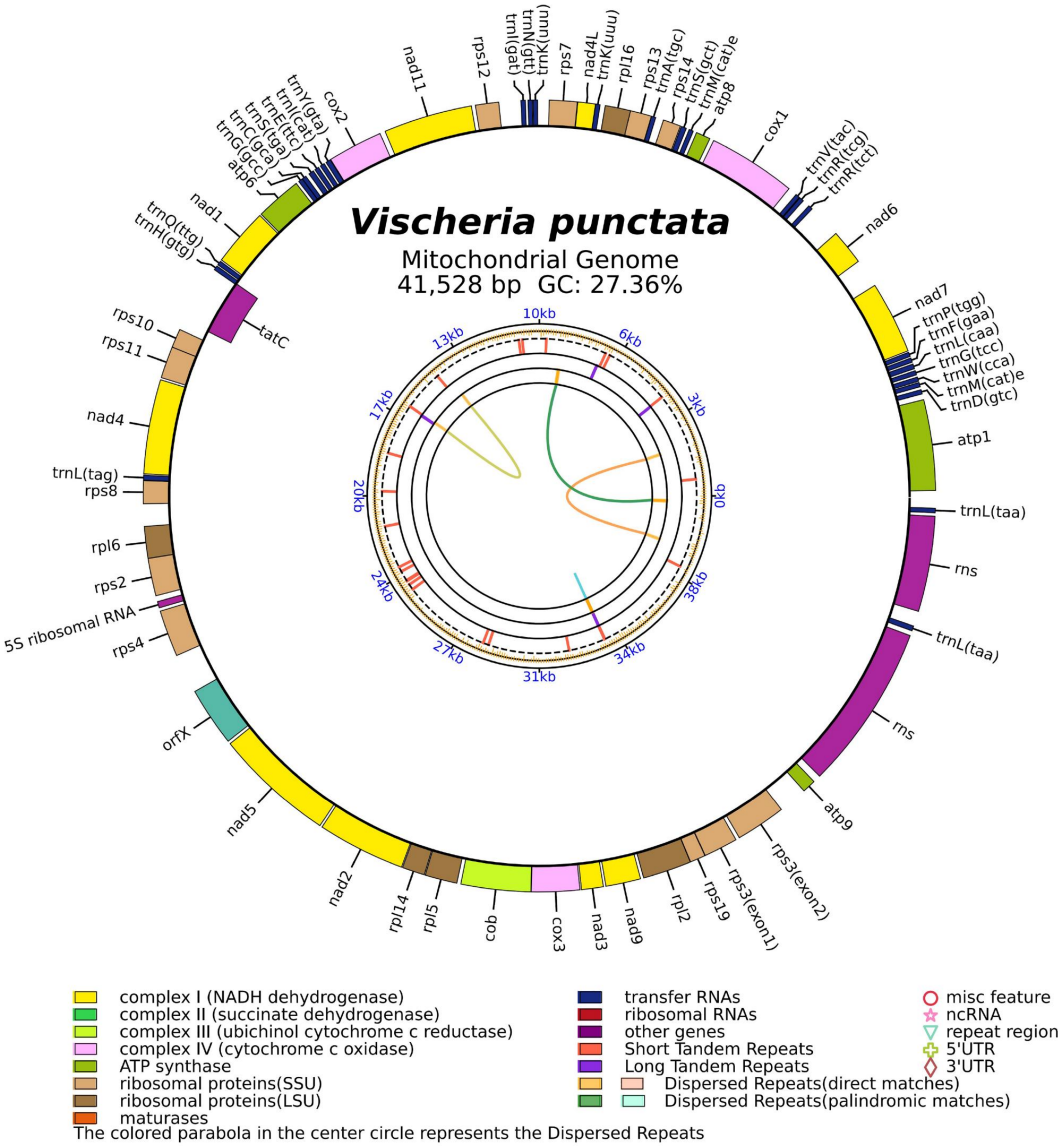
The circular map of the *V. punctata* mitochondrial genome. Genes belonging to different functional groups are color coded. Differences in colors lumps represent different gene types.

The total mitochondrial genome of *V. punctata*, a circular DNA molecule spanning 41,528 bp, has been duly archived in the NCBI database (GenBank accession no. MZ517137). The read coverage depth map is represented in Supplementary Figure S1, showcasing the minimum, maximum, and average read mapping depths of the assembled genome as 1841×, 3949×, and 2654×, respectively. This genome reveals a biased base composition of 39.2% adenine, 15.5% cytosine, 11.8% guanine, and 33.5% thymine. It encompasses three ribosomal RNA (rRNA) genes representing the 5S, small subunit, and large subunit, alongside 28 transfer RNA (tRNA) genes, and 36 protein-coding genes. These genes include four ATP genes, 16 ribosomal protein genes, 10 NADH genes, three cox genes, one apocytochrome b gene, one orfX gene, and one tatC gene (Figure 2). Interestingly, rps3 was the only gene identified as a cis-splicing gene, as depicted in Supplementary Figure S2.

**Figure 3.**
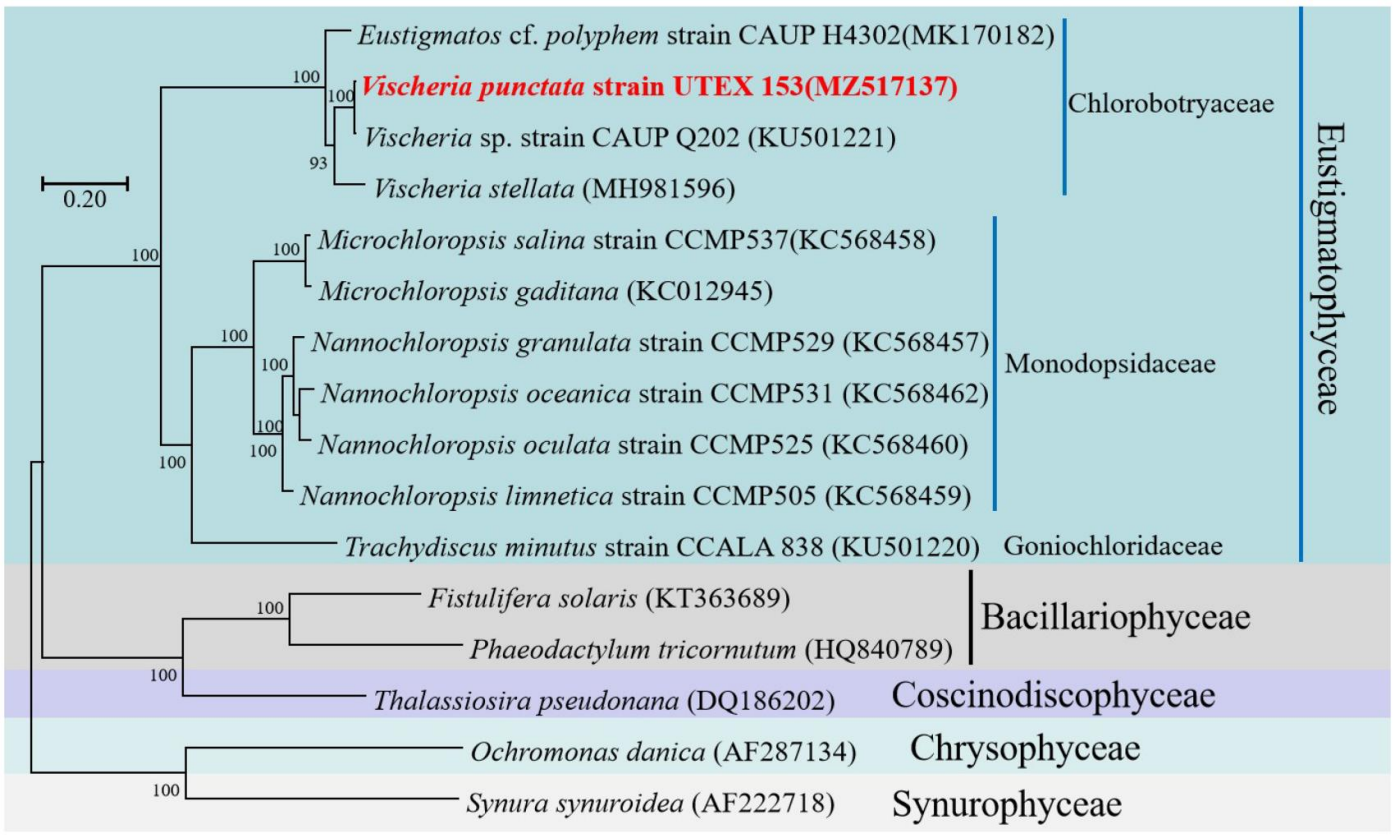
Phylogenetic relationships among 16 Ochrophyta mitochondrial genomes. Node labels indicate bootstrap values. The scale bar corresponds to 0.20 substitutions per nucleotide position. GenBank accession numbers for the utilized sequences are indicated within parentheses. *Eustigmatos* cf polyphem (MK170182) (Huang et al. 2018^#^), *Vischeria* sp. CAUP Q202 (KU501221) (Ševčíková et al. 2016), *Vischeria stellata* (MH981596) (Huang et al. 2019), *Microchloropsis salina* (KC568458) (Wei et al. 2013), *Microchloropsis gaditana* (KC012945), *Nannochloropsis granulata* (KC568457) (Wei et al. 2013), *Nannochloropsis oceanica* (KC568462) (Wei et al. 2013), *Nannochloropsis oculata* (KC568460) (Wei et al. 2013), *Nannochloropsis limnetica* (KC568459) (Wei et al. 2013), *Trachydiscus minutus* (KU501220) (Ševčíková et al. 2016), *Fistulifera solaris* (KT363689) (Tang and Bi 2016), *Phaeodactylum tricornutum* (HQ840789) (Oudot-Le and Green 2011), *Thalassiosira pseudonana* (DQ186202) (Armbrust et al. 2004), *Ochromonas danica* (AF287134) (Burger et al. 2016^#^), *Synura synuroidea* (AF222718) (Chesnick et al. 2000). ^#^Direct submission to NCBI, unpublished.

A phylogenetic analysis was performed to elucidate the evolutionary lineage of *V. punctata* (MZ517137) along with 15 other species within the Ochrophyta phylum. Based on the collective data of 33 genes, utilizing the GTR + F + I + G4 model, a phylogenetic tree was constructed (Figure 3). The 16 Ochrophyta species demonstrated considerable variation in size and were categorized into three prominent clades, encompassing the Classes of Eustigmatophyceae, Bacillariophyceae, and Chrysophyceae. It was noted that *V. punctata* clustered within the Chlorobotryaceae family in the *Vischeria* genus, forming a distinct clade with robust statistical support through posterior probabilities. The *Vischeria* genus was found to be in the closest proximity to the *Nannochloropsis* and *Microchloropsis* genera of the Monodopsidaceae family.

The Chlorobotryaceae family was recently redefined as one of the primary and most diverse lineages of the Eustigmataceae algae family, as per the findings of Barcytė et al. (2022). Eustigmataceae was established by Hibberd (1981) to accommodate the *Eustigmatos* and *Vischeria* genera. Given the negligible genetic variance between these two lineages, they have recently been merged into the single genus *Vischeria* (Kryvenda et al. 2018). Our study reaffirms that the mitochondrial genomes of *Eustigmatos* and *Vischeria* are closely related, substantiating *Eustigmatos* as a junior heterotypic synonym of *Vischeria*.

*V. punctata* was found to have a closer affiliation with *Vischeria* sp. CAUP Q202 than *Vischeria stellata*, suggesting phenotypic similarity. The collinearity to *Vischeria* sp. CAUP Q202 was significant (97% similarity), implying a common ancestor for *V. punctata* and *Vischeria* sp. CAUP Q202.

## Conclusions

We have effectively assembled the mitochondrial genome utilizing Illumina sequencing technology of *V. punctata*. Subsequently, a phylogenetic analysis was executed through the construction of a phylogenetic tree encompassing 16 species from the Ochrophyta phylum. This endeavor offers a novel approach to studying microalgae at the molecular level and furnishes pertinent information regarding evolutionary relationships within the Eustigmatophyceae class. To gain deeper insights into the phylogenetic associations among the Chlorobotryaceae species, it is necessary to broaden the study of mitochondrial genomes within this order. Future investigations may include assessments of collinear calculation, ancestral structural analyses, exploration of homologous recombination events, and estimates of divergence time pertaining to the *Vischeria* genus.

## Supplementary Material

Supplemental Material

Supplemental Material

## Data Availability

The genome sequence data that support the findings of this study are openly available in GenBank of NCBI under accession no. MZ517137.1. The associated BioProject, BioSample, and SRA numbers are PRJNA759399, SAMN21166780, and SRR15691269, respectively.

## References

[CIT0001] Abuduaini A, Wang YB, Zhou HY, Kang RP, Ding ML, Jiang Y, Suo FY, Huang LD. 2021. The complete mitochondrial genome of *Ophiocordyceps gracilis* and its comparison with related species. IMA Fungus. 12(1):31. doi:10.1186/s43008-021-00081-z. 34670626 PMC8527695

[CIT0002] Alverson AJ, Wei X, Rice DW, Stern DB, Barry K, Palmer JD. 2010. Insights into the evolution of mitochondrial genome size from complete sequences of *Citrullus lanatus* and *Cucurbita pepo* (Cucurbitaceae). Mol Biol Evol. 27(6):1436–1448. doi:10.1093/molbev/msq029. 20118192 PMC2877997

[CIT0003] Armbrust EV, Berges JA, Bowler C, Green BR, Martinez D, Putnam NH, Zhou S, Allen AE, Apt KE, Bechner M, et al. 2004. The genome of the diatom *Thalassiosira pseudonana*: ecology, evolution, and metabolism. Science. 306(5693):79–86. doi:10.1126/science.1101156. 15459382

[CIT0004] Bankevich A, Nurk S, Antipov D, Gurevich AA, Dvorkin M, Kulikov AS, Lesin VM, Nikolenko SI, Pham S, Prjibelski AD, et al. 2012. SPAdes: a new genome assembly algorithm and its applications to single-cell sequencing. J Comput Biol. 19(5):455–477. doi:10.1089/cmb.2012.0021. 22506599 PMC3342519

[CIT648346] Barcytė D, Zátopková M, Němcová Y, Richtář M, Yurchenko T, Jaške K, Fawley KP, Škaloud P, Ševčíková T, Fawley MW, et al. 2022. Redefining Chlorobotryaceae as one of the principal and most diverse lineages of eustigmatophyte algae. Mol Phylogenet Evol. 177:107607 doi:10.1016/j.ympev.2022.107607. 35963589

[CIT0005] Bernt M, Donath A, Jühling F, Externbrink F, Florentz C, Fritzsch G, Pütz J, Middendorf M, Stadler PF. 2013. MITOS: improved de novo metazoan mitochondrial genome annotation. Mol Phylogenet Evol. 69(2):313–319. doi:10.1016/j.ympev.2012.08.023. 22982435

[CIT0006] Bi G, Mao Y, Xing Q, Cao M. 2018. HomBlocks: a multiple-alignment construction pipeline for organelle phylogenomics based on locally collinear block searching. Genomics. 110(1):18–22. doi:10.1016/j.ygeno.2017.08.001. 28780378

[CIT0008] Chan PP, Lowe TM. 2019. tRNAscan-SE: searching for tRNA genes in genomic sequences. Methods Mol Biol. 1962:1–14. 31020551 10.1007/978-1-4939-9173-0_1 PMC6768409

[CIT0009] Chesnick JM, Goff M, Graham J, Ocampo C, Lang BF, Seif E, Burger G. 2000. The mitochondrial genome of the stramenopile alga *Chrysodidymus synuroideus*: complete sequence, gene content and genome organization. Nucleic Acids Res. 28(13):2512–2518. doi:10.1093/nar/28.13.2512. 10871400 PMC102714

[CIT0010] Dunn NA, Unni DR, Diesh C, Munoz-Torres M, Harris NL, Yao E, Rasche H, Holmes IH, Elsik CG, Lewis SE. 2019. Apollo: democratizing genome annotation. PLoS Comput Biol. 15(2):e1006790. doi:10.1371/journal.pcbi.1006790. 30726205 PMC6380598

[CIT0011] Eliáš M, Amaral R, Fawley KP, Fawley MW, Němcová Y, Neustupa J, Přibyl P, Santos LMA, Ševčíková T. 2017. Eustigmatophyceae. In: Handbook of the protists. Cham: Springer. Vol. 1; p. 367–406. doi:10.1007/978-3-319-28149-0_39

[CIT0012] Gao B, Xu M, Shan D, Zhang C, Yang Y, Dong Z, Zhang H, Han B, Huang L, Zhang C. 2023. The genomes of *Vischeria* oleaginous microalgae shed light on the molecular basis of hyper-accumulation of lipids. BMC Biol. 21(1):133. doi:10.1186/s12915-023-01618-x. 37280620 PMC10245428

[CIT0013] Gao B, Yang J, Lei X, Xia S, Li A, Zhang C. 2016. Characterization of cell structural change, growth, lipid accumulation, and pigment profile of a novel oleaginous microalga, *Vischeria stellata* (Eustigmatophyceae), cultured with different initial nitrate supplies. J Appl Phycol. 28(2):821–830. doi:10.1007/s10811-015-0626-1.

[CIT0015] Hibberd DJ. 1981. Notes on the taxonomy and nomenclature of the algal classes Eustigmatophyceae and Tribophyceae (synonym Xanthophyceae). Bot J Linn Soc. 82(2):93–119. doi:10.1111/j.1095-8339.1981.tb00954.x.

[CIT0016] Huang L, Gao B, Wang F, Zhang C. 2019. The complete mitochondrial genome of an oleaginous microalga *Vischeria stellata* strain SAG 33.83 (Eustigmatophyceae). Mitochondrial DNA Part B. 4(1):301–302. doi:10.1080/23802359.2018.1542993. PMC10798287 38249358

[CIT0017] Jagadevan S, Banerjee A, Banerjee C, Guria C, Tiwari R, Baweja M, Shukla P. 2018. Recent developments in synthetic biology and metabolic engineering in microalgae towards biofuel production. Biotechnol Biofuels. 11(1):185. doi:10.1186/s13068-018-1181-1. 29988523 PMC6026345

[CIT0018] Katoh K, Misawa K, Kuma K, Miyata T. 2002. MAFFT: a novel method for rapid multiple sequence alignment based on fast Fourier transform. Nucleic Acids Res. 30(14):3059–3066. doi:10.1093/nar/gkf436. 12136088 PMC135756

[CIT0019] Kryvenda A, Rybalka N, Wolf M, Friedl T. 2018. Species distinctions among closely related strains of Eustigmatophyceae (Stramenopiles) emphasizing ITS2 sequence-structure data: *Eustigmatos* and *Vischeria*. Eur J Phycol. 53(4):471–491. doi:10.1080/09670262.2018.1475015.

[CIT0020] Lagesen K, Hallin P, Rødland EA, Staerfeldt H-H, Rognes T, Ussery DW. 2007. RNAmmer: consistent and rapid annotation of ribosomal RNA genes. Nucleic Acids Res. 35(9):3100–3108. doi:10.1093/nar/gkm160. 17452365 PMC1888812

[CIT0021] Nguyen L-T, Schmidt HA, von Haeseler A, Minh BQ. 2015. IQ-TREE: a fast and effective stochastic algorithm for estimating maximum-likelihood phylogenies. Mol Biol Evol. 32(1):268–274. doi:10.1093/molbev/msu300. 25371430 PMC4271533

[CIT0022] Okonechnikov K, Golosova O, Fursov M. 2012. Unipro UGENE: a unified bioinformatics toolkit. Bioinformatics. 28(8):1166–1167. doi:10.1093/bioinformatics/bts091. 22368248

[CIT0023] Oudot-Le SMP, Green BR. 2011. Complex repeat structures and novel features in the mitochondrial genomes of the diatoms *Phaeodactylum tricornutum* and *Thalassiosira pseudonana*. Gene. 476(1–2):20–26. doi:10.1016/j.gene.2011.02.001. 21320580

[CIT0024] Ševčíková T, Klimeš V, Zbránková V, Strnad H, Hroudová M, Vlček Č, Eliáš M. 2016. A Comparative analysis of mitochondrial genomes in eustigmatophyte algae. Genome Biol Evol. 8(3):705–722. doi:10.1093/gbe/evw027. 26872774 PMC4824035

[CIT0025] She Y, Gao X, Jing X, Wang J, Dong Y, Cui J, Xue H, Li Z, Zhu D. 2022. Effects of nitrogen source and NaCl stress on oil production in *Vischeria* sp. WL1 (Eustigmatophyceae) isolated from dryland biological soil crusts in China. J Appl Phycol. 34(3):1281–1291. doi:10.1007/s10811-022-02720-3.

[CIT0026] Sinetova MA, Sidorov RA, Medvedeva AA, Starikov AY, Markelova AG, Allakhverdiev SI, Los DA. 2021. Effect of salt stress on physiological parameters of microalgae *Vischeria punctata* strain IPPAS H-242, a superproducer of eicosapentaenoic acid. J Biotechnol. 331:63–73. doi:10.1016/j.jbiotec.2021.03.001. 33727081

[CIT0027] Tang X, Bi G. 2016. Complete mitochondrial genome of *Fistulifera solaris* (Bacillariophycidae). Mitochondrial DNA A DNA Mapp Seq Anal. 27(6):4405–4406. doi:10.3109/19401736.2015.1089545. 26404767

[CIT0028] Tillich M, Lehwark P, Pellizzer T, Ulbricht-Jones ES, Fischer A, Bock R, Greiner S. 2017. GeSeq – versatile and accurate annotation of organelle genomes. Nucleic Acids Res. 45(W1):W6–W11. doi:10.1093/nar/gkx391. 28486635 PMC5570176

[CIT0029] Wang FF, Gao BY, Huang LD, Su M, Dai CM, Zhang CW. 2018. Evaluation of oleaginous eustigmatophycean microalgae as potential biorefinery feedstock for the production of palmitoleic acid and biodiesel. Bioresour Technol. 270:30–37. doi:10.1016/j.biortech.2018.09.016. 30212771

[CIT0030] Wei L, Xin Y, Wang D, Jing X, Zhou Q, Su X, Jia J, Ning K, Chen F, Hu Q, et al. 2013. *Nannochloropsis* plastid and mitochondrial phylogenomes reveal organelle diversification mechanism and intragenus phylotyping strategy in microalgae. BMC Genomics. 14(1):534. doi:10.1186/1471-2164-14-534. 23915326 PMC3750441

[CIT3730565] Wick RR, Schultz MB, Zobel J, Holt KE. 2015. Bandage: interactive visualization of de novo genome assemblies. Bioinformatics. 31(20):3350–3352. doi:10.1093/bioinformatics/btv383. 26099265 PMC4595904

[CIT0031] Yang HP, Wenzel M, Hauser DA, Nelson JM, Xu X, Eliáš M, Li FW. 2021. *Monodopsis* and *Vischeria* genomes shed new light on the biology of eustigmatophyte algae. Genome Biol Evol. 13(11):evab233. 34665222 10.1093/gbe/evab233 PMC8570151

[CIT0032] Zhang D, Gao F, Jakovlić I, Zou H, Zhang J, Li WX, Wang GT. 2020. PhyloSuite: an integrated and scalable desktop platform for streamlined molecular sequence data management and evolutionary phylogenetics studies. Mol Ecol Resour. 20(1):348–355. doi:10.1111/1755-0998.13096. 31599058

