# Supplementary material for: Complete mitochondrial genome of an oleaginous microalga Vischeria punctata (Eustigmatophyceae: Chlorobotryaceae) and phylogenetic analysis
Source: Mitochondrial DNA B Resour. 2024 Jan 18;9(1):94–9. doi: 10.1080/23802359.2023.2301027 (PMC10798287; doi:10.1080/23802359.2023.2301027)
Supplement: Supplemental Material [file TMDN_A_2301027_SM5022.pdf]

ZiboYimore Translation CO. LTD

To whom it may concern:The purpose of this letter is to verify that Zibo Yimore Translation CO. LTD provided the English proofreading services for the following thesis : Complete mitochondrial genome of an oleaginous microalga *Vischeria punctata* (Eustigmatophyceae: Chlorobotryaceae) and phylogenetic analysis

Author: Zhouwei Luo, Yanhang Tang, Zihao Wang, Yuexin Sun, Yu Jiang, Wenjie Yang, Ge Chen, and Luodong Huang

The basic language editing including the correction of grammar, punctuation and syntax was performed normatively with our best efforts. And the edited document was returned to the writer on 08/08/2023. We are unaware of any changes or additions made to the manuscript after that time. We have kept the specimen of the received original file in case of any legal dispute.

Sincerely,

Henry Graff

Zibo Yimore Translation CO. LTD

Tel: 0533-2775538

Address: Meishistreet NO.137, Zhangdian District, Zibo city, Shandong Province
