# Supplementary material for: Complete mitochondrial genome of an oleaginous microalga Vischeria punctata (Eustigmatophyceae: Chlorobotryaceae) and phylogenetic analysis
Source: Mitochondrial DNA B Resour. 2024 Jan 18;9(1):94–9. doi: 10.1080/23802359.2023.2301027 (PMC10798287; doi:10.1080/23802359.2023.2301027)
Supplement: Supplemental Material [file TMDN_A_2301027_SM4992.doc]

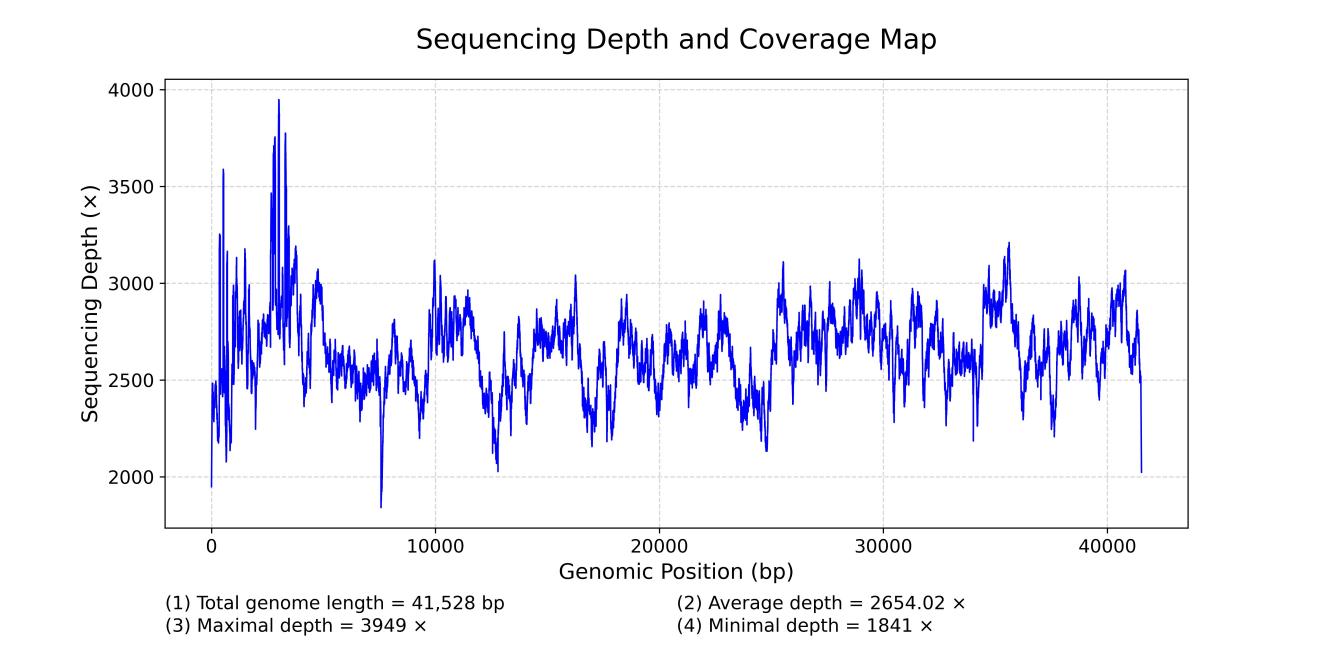


**Supplementary Figure S1.** Generating sequencing depth and coverage map of mitogenome sequence of *V. punctata*.


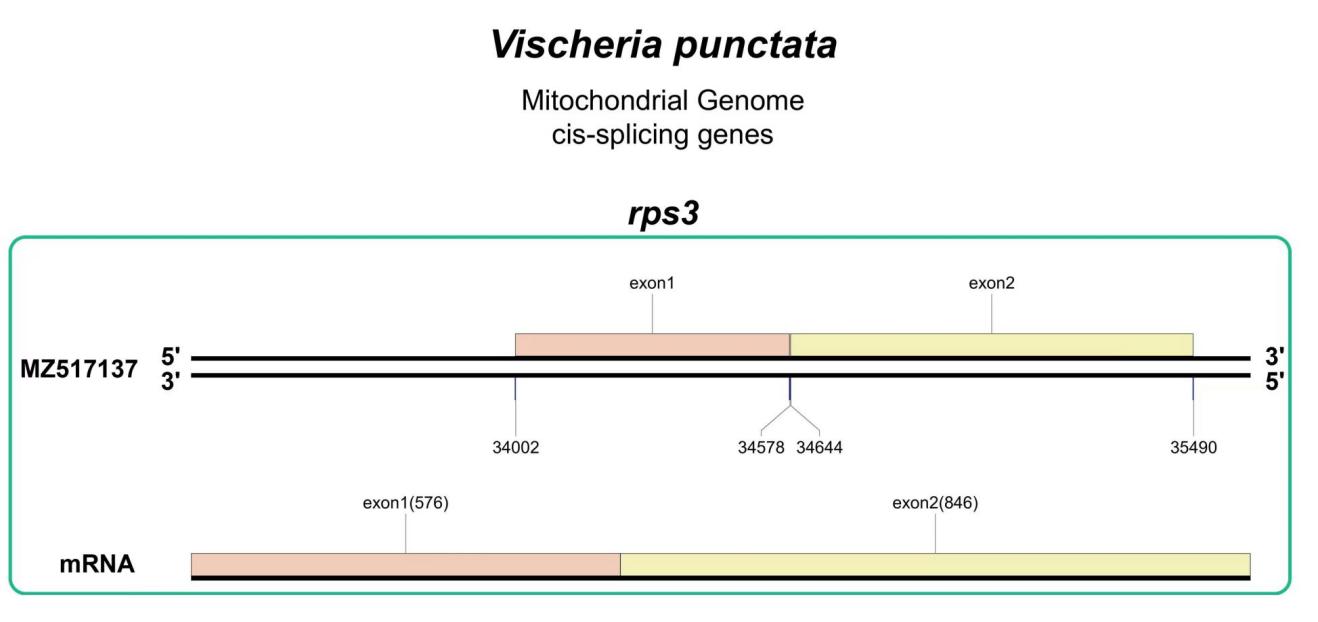


**Supplementary** **Figure S2.** This cis-splicing gene images for mitogenome of *V. punctata.*
